# Co-editing *PINK1* and *DJ-1* Genes *Via* Adeno-Associated Virus-Delivered CRISPR/Cas9 System in Adult Monkey Brain Elicits Classical Parkinsonian Phenotype

**DOI:** 10.1007/s12264-021-00732-6

**Published:** 2021-06-24

**Authors:** Hao Li, Shihao Wu, Xia Ma, Xiao Li, Tianlin Cheng, Zhifang Chen, Jing Wu, Longbao Lv, Ling Li, Liqi Xu, Wenchao Wang, Yingzhou Hu, Haisong Jiang, Yong Yin, Zilong Qiu, Xintian Hu

**Affiliations:** 1 Key Laboratory of Animal Models and Human Disease Mechanisms of the Chinese Academy of Sciences and Yunnan Province, Kunming Institute of Zoology, Chinese Academy of Sciences, 650223 Kunming, China; 2 Institute of Neuroscience, CAS Key Laboratory of Primate Neurobiology, State Key Laboratory of Neuroscience, Chinese Academy of Sciences, Shanghai, 200031 China; 3 Institutes of Physical Science and Information Technology, Anhui University, Hefei, 230601 China; 4 Center for Excellence in Brain Science and Intelligence Technology, Chinese Academy of Sciences, Shanghai, 200031 China; 5 National Resource Center for Non-human Primates, Kunming Primate Research Center, and National Research Facility for Phenotypic and Genetic Analysis of Model Animals (Primate Facility), Kunming Institute of Zoology, Chinese Academy of Sciences, 650107 Kunming, China; 6 Diagnostic Radiology Department, 920 Hospital of the Joint Logistics Support Force of the PLA, Kunming, 650032 China; 7 Ultrasound diagnosis Department, 920 Hospital of the Joint Logistics Support Force of the PLA, Kunming, 650032 China; 8 Sichuan Provincial People’s Hospital, University of Electronic Science and Technology of China, Chengdu, 610072 China; 9 Department of Rehabilitation Medicine, The Second People’s Hospital of Yunnan Province, Kunming, 650021 China; 10 National Clinical Research Center for Aging and Medicine, Huashan Hospital, Fudan University, Shanghai, 200433 China

**Keywords:** Parkinson’s disease, Monkey, Adeno-associated virus-delivered CRISPR/Cas9, *PINK1*, *DJ-1*, Parkinsonian phenotype

## Abstract

Whether direct manipulation of Parkinson’s disease (PD) risk genes in the adult monkey brain can elicit a Parkinsonian phenotype remains an unsolved issue. Here, we used an adeno-associated virus serotype 9 (AAV9)-delivered CRISPR/Cas9 system to directly co-edit *PINK1* and *DJ-1* genes in the substantia nigras (SNs) of two monkey groups: an old group and a middle-aged group. After the operation, the old group exhibited all the classic PD symptoms, including bradykinesia, tremor, and postural instability, accompanied by key pathological hallmarks of PD, such as severe nigral dopaminergic neuron loss (>64%) and evident α-synuclein pathology in the gene-edited SN. In contrast, the phenotype of their middle-aged counterparts, which also showed clear PD symptoms and pathological hallmarks, were less severe. In addition to the higher final total PD scores and more severe pathological changes, the old group were also more susceptible to gene editing by showing a faster process of PD progression. These results suggested that both genetic and aging factors played important roles in the development of PD in the monkeys. Taken together, this system can effectively develop a large number of genetically-edited PD monkeys in a short time (6–10 months), and thus provides a practical transgenic monkey model for future PD studies.

**Supplementary Information:**

The online version contains supplementary material available at 10.1007/s12264-021-00732-6.

## Introduction

Parkinson’s disease (PD), with a prevalence of 2%–3% in people >65 years old, is the second-most common neurodegenerative disorder worldwide [1]. It is typically characterized by a cluster of specific motor symptoms, including bradykinesia, rigidity, postural instability and tremor, and by a set of unique pathological hallmarks, including severe dopaminergic neuron loss and clear morphological changes of the surviving dopaminergic neurons in the substantia nigra pars compacta (SNpc), and intracellular inclusions containing α-synuclein aggregates, which are named Lewy bodies in neuronal somata or Lewy neurites in neuronal processes in the SN and other brain regions [1, 2]. The severe dopaminergic neuronal loss and related morphological changes that appear in every PD patient are the most important pathological hallmarks of PD [3, 4]. Lewy bodies appear in 77%–95% of patients according to different studies, and this is the second most important hallmark [4, 5]. Currently, limited by its unclear etiology, no effective treatment to alleviate PD progression and no preventative measures are available [6], and there is a significant increase of PD patients every year (approximately 0.02%) [7]. Solving this ongoing crisis by the development of early diagnosis and prevention has become a consensus in the field. Models of PD etiology, such as transgenic models that mimic the development of PD, are the most important foundation to achieve this goal. Unfortunately, no breakthroughs have been reported in this area yet.

In addition to developing models that mimic the etiology of PD, selecting the proper animals for modeling is equally important. Non-human primates are ideal experimental animals in which to study the etiology and pathogenesis of PD due to their evolutionary proximity, physiological similarity, close aging process, equivalent behavioral symptoms, and pathological changes to those in PD patients [8, 9]. More importantly, our recent study showed that monkeys can suffer from PD naturally [10], suggesting that monkeys share this disease with humans and have the potential to closely model the developmental processes in PD patients.

The existing classic monkey PD models, which are induced by the administration of neurotoxins such as 1-methyl-4-phenyl-1, 2, 3, 6-tetrahydropyridine (MPTP) or 6-hydroxydopamine, only mimic the symptoms and some of the pathological hallmarks of PD. Since the neurotoxins are mainly man-made and do not exist in nature, the neurotoxin-induced models can hardly reflect the real process of PD development in patients. On the other hand, a gene-editing PD monkey model established by changing its genes to mimic the PD risk mutations discovered in humans, is an etiological model that can be used to study the pathogenesis and screen for early diagnostic biomarkers of PD [11]. To date, however, no successful transgenic PD monkeys have been reported, despite decades of research recapitulating the genetic factors involved in PD etiology and pathogenesis [3, 12]. Nevertheless, recent progress in gene-editing technology heralds a new era in the development of genetically-manipulated PD monkeys [13–19], especially the clustered regularly interspaced short palindromic repeat/CRISPR-associated protein 9 (CRISPR/Cas9) system, which allows sequence-specific gene-editing in many organisms and provides a valuable tool to generate gene-edited animal models of human diseases [20–23].

It is well known that mutations in PD risk genes are the primary causes of neurodegeneration in patients with early-onset PD [3]. Notably, the *PINK1/PARK6* gene is an autosomal recessive PD risk gene implicated in mitochondrial functions, and *PINK1* mutations account for 1%–7% of early-onset PD cases [24]. One critical function of the PINK1 protein is the phosphorylation of Parkin protein (an E3 ubiquitin ligase), which is important for protein degradation in proteasomes [12, 24]. However, loss-of-function mutations of *PINK1 in vivo* do not elicit the clinical symptoms of PD in monkeys [17], and our data further revealed that editing the *PINK1* gene *via* the AAV9-delivered CRISPR/Cas9 system in the SN regions of adult monkeys only causes approximately 40% nigral dopaminergic cell loss (Fig. S1A, B). Based on our and others’ previous studies, it is well known that the loss of nigral dopaminergic neurons has to be at least 50% in one or both SNs to elicit PD symptoms [25, 26]. Therefore, *PINK1* gene mutation alone may not be enough to trigger the development of PD symptoms in monkeys. Another autosomal recessive PD risk gene, *DJ-1/PARK7*, which encodes an antioxidant protein, potentially works with *PINK1* to inhibit oxidative stress and α-synuclein aggregation in the pathogenesis of PD [27, 28]. A second experiment, in which the *DJ-1* gene in the SN regions of adult monkeys was also edited *via* the same system, was carried out in our lab. The data showed that this procedure did not elicit PD clinical symptoms either, for it also caused only approximately 40% nigral dopaminergic cell loss (Fig. S1C, D).

In this study, based on the two sets of preliminary data (Fig. S1), we used the AAV9-delivered CRISPR/Cas9 system to co-edit the *PINK1* and *DJ-1* genes in the SN region of the monkey brain to investigate the possibility that classical PD symptoms might be elicited by this double-gene-editing strategy.

## Materials and Methods

### Animal Ethics

All experiment protocols and animal welfare were approved (No. IACUC15001) by the Ethics Committee of Kunming Primate Research Center (AAALAC accredited), and Kunming Institute of Zoology, the Chinese Academy of Sciences. Animals in this study were also treated in accordance with the National Institutes of Health (USA) Guide for the Care and Use of Laboratory Animals (8^th^ edition, 2011; National Academies Press, Washington, DC), and the ARRIVE guidelines for reporting animal research [29]. The monkeys were cared for by veterinarians and fed under standard procedures in individual cages with a standardized light/dark cycle. Best efforts were made to minimize their suffering and the number of monkeys used in this study.

A total of 10 adult male rhesus monkeys (*Macaca mulatta*) from Kunming Primate Research Center were included in the study. Four were involved in a preliminary study in which the *PINK1* or *DJ-1* gene was edited alone. Another four were included in the current study in which both the *PINK1* and *DJ-1* genes were edited together. The remaining two monkeys were age-matched normal controls (without any procedures) for tyrosine hydroxylase (TH) immunostaining used in our previous studies [26, 30]. Detailed information for all the monkeys is listed in Table 1.

**Table 1:** Detailed information on the monkeys involved in this study

Identification (no.)	Age (years)^a^	Gender	Experimental treatment
070229	10	Male	Left SN: *AAV9-SaCas9-SgPINK1+DJ-1*
070209	10	Male	Right SN: *AAV9-SaCas9-SgPINK1+DJ-1*
94089	23	Male	Left SN: *AAV9-SaCas9-SgPINK1+DJ-1*
97093	21	Male	Right SN: *AAV9-SaCas9-SgPINK1+DJ-1*
90067	26	Male	Left SN: *AAV9-SaCas9-SgPINK1*
95301	21	Male	Left SN: *AAV9-SaCas9-SgPINK1*
95075	21	Male	Left SN: *AAV9-SaCas9-SgDJ-1*
97081	19	Male	Left SN: *AAV9-SaCas9-SgDJ-1*
99357 (#14) [26]	14	Male	Normal control: age-matched for 070229
91097 [30]	24	Male	Normal control: age-matched for 94089

^a^Indicates age at death.

### Viral Vectors

The AAV9-SaCas9 vector was from pX602-AAV-TBG::NLS-SaCas9-NLS-HA-OLLAS-bGHpA;U6::BsaI-sgRNA (#61593, Addgene, Watertown, MA, USA). The Thyroxine Binding Globulin (TBG) promoter was replaced with human synapsin promoter for better expression in the brain. The single-guide RNAs (sgRNAs) were annealed from DNA oligos with BsaI digest ends. After annealing, the sgRNA segments were inserted into the BsaI site of the vector. The viral titers were as follows: vehicle-control: 4.00 × 10^12^ vg/mL; *PINK1*-A: 7.00 × 10^12^ vg/mL; *PINK1*-B: 9.00 × 10^12^ vg/mL; *DJ-1-*A: 1.20 × 10^13^ vg/mL; *DJ-1-*B: 8.00 × 10^12^ vg/mL.

### Mutation Efficiency Assessment in the COS7 Cell Line

The COS7 cell line was cultured in Dulbecco’s Modified Eagle Medium (DMEM) high glucose (Thermo Fisher Scientific, Waltham, MA, USA) with 10% fetal bovine serum in 37 °C, 5% CO_2_, and passaged every 3–4 days. In order to assess the efficiency of sgRNAs, sgRNAs and anti-puromycin vectors were transfected into COS7 cells plated in 6-well plates before transfection. When the coverage reached 80%, 6 mg plasmid (4 mg sgRNA vector, 2 mg anti-puro vector) with 4 mg Lipofectamine 3000 was added to each well. After 48 h, puromycin was added to a final concentration in the medium of 10 mg/mL to screen out negative cells. Surviving cells were collected after 24 h, digested in lysis buffer with 0.4 mg/mL protease K, and the genome was extracted with phenol chloroform. The regions including the sgRNA target sites were amplified by polymerase chain reaction (PCR), and then the PCR products were ligated to T vector by T4 ligase after purification. By transforming competent *E. coli*, positive monoclones were selected for sequencing. The results were compared with wild-type control sequencing results to determine the efficiency of sgRNA.

Primers used in the PCR amplification were:

- *PINK1*-A forward: 5’-CCTGATCTTACCCACTTGC-3’
- *PINK1*-A reverse: 5’-GCTCCTGCTCTTCTCCTG-3’
- *PINK1*-B forward: 5’- TCACCTTGGCATCTCCTC-3’
- *PINK1*-B reverse: 5’-GCTCTACCCGTGCATTTC-3’
- *DJ-1*-A forward: 5’-ACAGGTTAATTGCGAAGG-3’
- *DJ-1*-A reverse: 5’-GTGGGAAGATGGTTTGAG-3’
- *DJ-1*-B forward: 5’-CAGGAAGGAGATTATACTACC-3’
- *DJ-1*-B reverse: 5’-CAATAGAACACAAGCAGATG-3’

### Surgery

#### Magnetic Resonance Imaging (MRI)-Guided SN Localization

Accurate localization (error <1 mm) of the monkey SNs was a prerequisite for effective AAV9-based gene editing, because the AAV9 editing system had to be injected to cover the whole of both SNs. We used an MRI-guided SN localization procedure developed in our lab that had been successfully used to accurately locate >30 SNs in monkeys’ brains in our previous PD experiments and related study [26]. Before the localization process, the monkey was intramuscularly (i.m.) injected with atropine (1 mL, 0.5 mg/mL; Handu, Xinzheng, China). After 10 min, the monkey was first anesthetized with ketamine (1.0–1.5 mL, 0.1 g/2 mL, i.m.; Zhongmu, Taizhou, China) and then injected with pentobarbital sodium (40 mg/mL, 20 mg/kg, i.m.; Fluka, Germany) for prolonged surgical anesthesia.

**Fig. 1:**
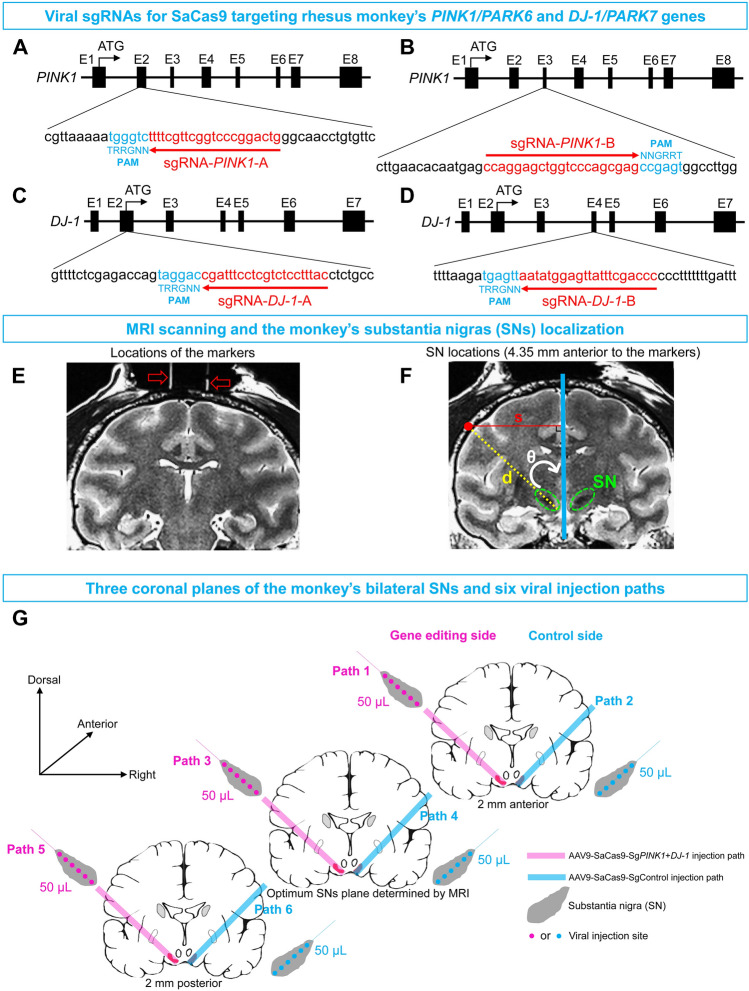
Design of sgRNAs for the SaCas9 gene-editing systems and the viral injection paths for the substantia nigras (SNs) in the brain of monkey Old 1 that based on an MRI localization procedure. **A–D** SaCas9-corresponding sgRNAs designed for monkey *PINK1/PARK6* and *DJ-1/PARK7* genes. The targeting sites of *PINK1* (**A** and **B**) and *DJ-1* (**C** and **D**) genes in rhesus monkey genome are shown, in which the sgRNA-binding sequences are highlighted in red and PAM sequences are marked in blue. Abbreviations: E1–8, exons 1–8; ATG: adenine-thymine-guanine; PAM, protospacer adjacent motif. **E** Image of the two markers (glass tubes filled with glycerin indicated by the red arrows) implanted in the skull of monkey monkey  Old 1 and used as references to localize the SNs. **F** Image of the locations of the bilateral SNs (outlined in green) in monkey Old 1 and an injection path (yellow dashed line) on one side for gene-editing are illustrated (blue line, axis of symmetry in coronal brain section; red dot, skull site for viral injection; s, horizontal distance from red dot to blue line; d, distance from skull to the most medial part of the SN; θ, angle between the yellow dashed line and blue line). The location of the red dot is accurately determined by s and θ. **G** Schematic showing of three parallel paths (pink lines 1, 3, and 5) of AAV9-SaCas9-Sg*PINK1*+*DJ-1* injections containing 18 injection sites that cover the left SN, and three parallel paths (blue lines 2, 4, and 6) of AAV9-SaCas9-SgControl injections containing 18 injection sites.

In the MRI-guided SN localization procedure, the first step was to determine the coronal section closest to the middle of the SN. To achieve this, two markers were fixed to the skull to serve as references. Their coordinates were 10 mm anterior the ear bar zero and a medial lateral distance of 5 mm to the left and right of the midsagittal plane. The depth of both was 1 mm into the skull. These coordinates were determined from the Rhesus Monkey Brain Atlas (The Rhesus Monkey Brain in Stereotaxic Coordinates, 1999, Academic Press, San Diego, page 163). The markers used here were two glass tubes filled with glycerin (3 cm long; internal diameter, 0.5 mm; external diameter, 0.9 mm), which appeared as clear high-intensity signals in the MRI T2 images (Magnetom Verio 3T, Siemens, Erlangen, Germany). Details of the marker fixation and MRI scanning for the deep brain structure localization procedure are described in our previous report [31]. The markers appeared as two white parallel lines in the middle of a coronal section of the MRI image (Fig. 1E).

Next step was to determine the anterior/posterior coordinates of the SNs. The SNs were clearly seen on T2 images due to their low-intensity signal, appearing black in the MR image (Fig. 1F, green circles). Anatomically, the coronal section with the clearest morphology and largest area of SN was the closest to the middle of the SN from its most anterior end to its most posterior end. Hence this was selected as the optimum coronal section to calculate the coordinates for the injection paths (Fig. 1F). The distance from this section to the coronal section with the markers varied among monkeys. For example, the SNs of monkey Old 1 were 4.35 mm anterior to the section with the markers (Fig. 1F). From this optimal coronal section, it was clear that the three parameters needed to determine the injection path in this section were “θ”, “s”, and “d”. In this optimal section, the axis of symmetry of the brain was marked with a blue line, and the path through the middle of the left SN was represented with a yellow dashed line. The distance from the most medial SN to the skull was “d”. The intersection of the yellow dashed line with the skull was marked with a red dot. The length of the horizontal line from this red dot to the blue line was “s”. “θ” was the angle between the blue line and the yellow dashed line. All three parameters: “θ”, “s”, and “d” were measured using the MRI software (Syngo MR B17, Siemens, Erlangen, Germany). To achieve maximum coverage of the targeted SN, two other paths with the same parameters were added in two coronal planes located 2 mm anterior and 2 mm posterior to the optimum SN plane. Thus, a total of three parallel injection paths were used for each SN (Fig. 1G). All the parameters for the viral injections of the four monkeys are listed in Table 2. When the MRI scans were completed, the markers were removed from the skull, the wounds were sutured, and the monkeys were given penicillin (800 kunit, i.m.; H13021634, Shiyao, Shijiazhuang, China) daily for one week.

**Table 2:** MRI-based coordinates of the viral injection paths for each monkey.

Monkey	Coordinates of bilateral SNs for viral injections						
	AAV9-SaCas9-Sg*PINK1*+*DJ-1* side				AAV9-SaCas9-SgControl side		
Middle age 1: 070229	Path	Path 1: −6.80	Path 3: −4.80	Path 5: −2.80	Path 2: −6.80	Path 4: −4.80	Path 6: −2.80
	s	+27.20	+27.20	+27.20	−24.60	−24.60	−24.60
	d	−36.70	−36.70	−36.70	−34.50	−34.50	−34.50
	θ	37°	37°	37°	35°	35°	35°
Middle age 2: 070209	Path	Path 1: −6.40	Path 3: −4.40	Path 5: −2.40	Path 2: −6.40	Path 4: −4.40	Path 6: −2.40
	s	−26.00	−26.00	−26.00	+25.70	+25.70	+25.70
	d	−35.00	−35.00	−35.00	−36.00	−36.00	−36.00
	θ	41°	41°	41°	38°	38°	38°
Old 1: 94089	Path	Path 1: −6.35	Path 3: −4.35	Path 5: −2.35	Path 2: −6.35	Path 4: −4.35	Path 6: −2.35
	s	+24.70	+24.70	+24.70	−24.80	−24.80	−24.80
	d	−36.80	−36.80	−36.80	−37.00	−37.00	−37.00
	θ	37°	37°	37°	37°	37°	37°
Old 2: 97093	Path	Path 1: −2.00	Path 3: 0	Path 5: 2.00	Path 2: −2.00	Path 4: 0	Path 6: 2.00
	s	−23.90	−23.90	−23.90	+25.20	+25.20	+25.20
	d	−38.10	−38.10	−38.10	−38.00	−38.00	−38.00
	θ	35°	35°	35°	37°	37°	37°

Coordinate description: Path: anterior (−) or posterior (+) to the plane with the markers (mm), **s**: left (+) or right (−) of the midsagittal plane (mm), **d**: distance (−) from the skull surface  to the most medial SN (mm), **θ**: angle between the injection path and the axis of symmetry of brain (°).

#### Viral Injections

After recovery for one week, each monkey underwent another operation for the viral injections and the anesthesia was as above. The coordinates for each injection path are listed in Table 2, and a small hole (1 mm in diameter) was drilled at the corresponding spot on the skull (Fig. 1F, red point). When the virus-loaded micro-injector reached the endpoint of the most medial side of the targeted SN, a multi-point injection strategy was adopted to ensure maximum coverage of the SN. In total, 6 sites at 1-mm intervals along each path were arranged for viral injection (50 μL in total per path). The injections of the other 2 paths for the same SN were carried out in the same way. Thus, a total of three parallel injection paths and 18 injection sites of a total of 150 μL virus completed the injection process (Fig. 1G). The administration speed was held constant at 800 nL/min by an injection pump (UMP 3-4, World Precision Instruments, Inc., USA). After the injections, the wound was sutured. All monkeys were given penicillin (800 kunit, i.m.; H13021634, Shiyao, Shijiazhuang, China) daily for one week.

### Behavioral Data Collection and Analyses

To evaluate the Parkinsonian behavior of the monkeys and dysfunctions of the PD-related SN-striatum pathway, we collected and analyzed behavioral data from three aspects: (1) quantification of the PD symptoms by videotaping the monkey’s daily activities and then scoring them using the improved version of the Kurlan scale (a Monkey-Parkinsonism Rating Scale); (2) measurement of deficits in SN-striatum pathway functions by the apomorphine (Apo)-induced rotation behavioral test; and (3) measurement of deficits of fine movement by a food-grasping task.

#### Quantification of the PD Symptoms

To identify and quantify the development of PD symptoms in the gene-edited monkeys, video recordings were essential. The video recordings of each monkey’s behavior were collected by digital cameras (Sony HDR-XR260, Japan). Briefly, a single-caged monkey was recorded without external disturbance for 1 h by a camera located 1 m in front of the cage from 10:00 to 11:00. After behavioral data collection, the 1-h video was used for the behavioral assessment. During the experiment, this behavioral data was collected a total of 7 times: on each Wednesday morning of weeks 0 (baseline), 6, 10, 12, 14, 16, and 18 after the viral injections.

The next step was video analysis based on previous studies [26, 32]. Two well-trained observers analyzed the video clips. The videos were randomly labeled by the experimenter with the letters from A to D each time. Blind to the conditions or treatments of each monkey, the two observers individually assigned scores to the video clips using the improved Kurlan scale.

The improved Kurlan scale is a widely-accepted standard scale for PD research on Old World monkeys [33]. It includes 4 parts: part A: Parkinsonian features; part B: drug-related side-effects; part C: overall level of activity; part D: clinical staging [34]. The PD score is calculated from part A, which includes seven PD measurements: tremor, posture, gait, bradykinesia, balance, gross motor skill, and defense reaction [34]. With a total possible PD score of 20, the score of each of the seven items in part A is evaluated and assigned by the severity (tremor, 0–3; posture, 0–2; gait, 0–4; bradykinesia, 0–4; balance, 0–2; gross motor skill, 0–3; defense reaction, 0–2). The total PD score is the sum of each score (see Table S1 for details).

After the scoring if there were no marked differences (<2 points) between the observers in evaluating each item of the scale, the data were pooled for creating figures and performing statistics; otherwise, the scores were checked by the experimenter and the final score was determined by consensus after watching the video together.

#### Apo-induced Rotational Behavioral Test

To measure the deficits in the function of the nigrostriatal dopaminergic system caused by *PINK1* and *DJ-1* editing, we used the classic pharmacological test, Apo administration-induced rotation. This rotation test consisted of two steps: first, the number of the spontaneous rotations to the lesioned side of each monkey was counted in a 30-min episode from the 15^th^ to the 45^th^ min of the whole 1-h video as above in daily behavioral recording. Second, a new video of the Apo-induced rotations to the healthy side was taken 10 min after the monkey was injected with Apo. Evaluation of the rotations was also based on a 30-min section from the 15th to the 45th min of the 1-h video after Apo administration. In the test, 2 mg of Apo was dissolved (1 mg/mL) in vitamin C solution (0.5 mg/2 mL) 5 min before the injection (2 mL, i.m.). During the experiment, the rotational behavioral tests were carried out 5 times on each Wednesday of weeks 0 (baseline), 6, 10, 12, and 14 after the viral injections following the behavioral video recording. This test was terminated at week 14 after the viral injections because the general condition of monkey Old 1 (94089) became very poor due to the severity of the PD symptoms and it was sacrificed for pathological study. All the videos were randomly labeled by the experimenter with numbers from 1 to 8 each time. Blind to the conditions or treatments of the monkeys, two observers individually counted the rotations in all the videos.

#### Food-Grasping Task

A food-grasping task was used to study the deficits in refined movement of each monkey during the development of PD [35]. In the task, the individually-caged monkey was trained to fetch a piece of food as a reward from a food box in front of its cage. After the fetching behavior stabilized, baseline data were collected. The latency, accuracy of food-grasping, and the hand preference were recorded. Each monkey had to finish 180 trials over three successive days (60 trials per day) for each testing week. Peanuts, diced apples, or food pellets were selected as rewards depending on each monkey’s preference. During the experiment, the food-grasping task was carried out 5 times, on weeks 0 (baseline), 6, 10, 12, and 14 after the viral injections. This test also ended at week 14 because of the generally poor condition of monkey Old 1 (94089).

### Validation of Pathological PD Hallmarks, Gene-Editing Effect Test, and Glial Activation Study

#### Treatment of Brain Tissue for the Pathological PD Hallmark Study

After the behavioral data collection, seven monkeys were sacrificed for pathological study. The seven monkeys included four involved in our preliminary study of the *PINK1* or *DJ-1* single-gene editing, and three involved in the current study on the *PINK1* and *DJ-1* gene co-editing. Among the latter three monkeys, the general condition of monkey Old 1 (94089) became very poor because of the severity of the PD symptoms at week 14 after viral injections, and it was sacrificed at an earlier time than the other monkey (Middle age 1: 070229, sacrificed at week 20 after viral injections). Monkey Middle age 2 (070209) was also sacrificed for pathological studies at week 20 after viral injections. The remaining monkey (Old 2: 97093) was still under long-term observation. Data from the additional two age-matched control monkeys (without any procedures) from our previous studies [26, 30] were pooled in this data set, serving as normal controls in the analyses.

These seven monkeys were first anesthetized with ketamine (2 mL, 0.1 g/2 mL, i.m.; Zhongmu, Taizhou, China) and then given pentobarbital sodium (40 mg/mL, 20 mg/kg, i.m.; Fluka, Germany). Perfusion was carried out after anesthesia. Each monkey’s brain was removed and fixed in 4% paraformaldehyde (PFA)-0.01 mol/L PBS for one week and then gradually equilibrated with 20% and 30% sucrose except for the monkey Middle age 2 (described below). Coronal sections (20 μm) of the whole SNs were cut on a freezing microtome (Leica, CM1850UV-1-1, Germany) at −24 °C.

#### Treatment of Brain Tissue from Monkey Middle Age 2 for Gene-Editing Effect and Glial Activation Study

The brain of Middle age 2 (070209) was cut into small blocks after perfusion, fixed with 4% PFA in phosphate buffer (0.01 mol/L PBS), and then equilibrated in 30% sucrose. The fixed and equilibrated blocks of the SN region were cut into 50-μm coronal sections on a Microm HM525 cryostat for further immunofluorescence co-staining of PINK1/Paris, DJ-1, GFAP, and Iba1 combined with EGFP.

#### Determination of the Anterior-Posterior (AP) Length of the SNs

The most important brain area involved in PD pathology is the SN, a paired flat structure located deep in the narrow mesencephalon [26, 36]. It was necessary to determine the AP length of the whole SN before sectioning. The length of the rhesus monkey SN was determined by reference to “The Rhesus Monkey Brain in Stereotaxic Coordinates, 1999, Academic Press, San Diego” and then verified by a set of scanning data from rhesus monkeys on a 3T MRI (Magnetom Verio, Siemens, Erlangen, Germany).

First, according to the monkey brain atlas, the SN of the rhesus monkey is located from −9.90 mm to −17.78 mm AP from bregma. As a result, its AP length is 7.88 mm. Second, using MRI data, the AP length of the rhesus monkeys’ SNs was measured and ranged from 6.8–8.0 mm. To ensure inclusion of the whole SN, the total AP length of a rhesus monkey SN was defined as 8.0 mm.

#### Cutting SN Sections for Immunostaining

The total AP length of the SN was defined as 8.0 mm by the above procedure. After this, the SN was cut into coronal sections at 20 μm. The interval between every two sections was 80 μm, and, as a result, ~80 sections were obtained from each SN. All the SNs were cut in the same way.

Next, we selected the optimal sections of the cut SN for immunostaining. The anatomical criteria for selecting the optimal portion of the SN for pathological study was established by Chu *et al*. [37–39], Gundersen *et al*. [40], and Dickson *et al*. [36]. The criterion is to select the portion that overlaps the rootlets of the third cranial nerve (CN III). The SN is in the ventral midbrain and defined ventrally by the cerebral peduncle and medially by the CN III rootlets in coronal section [36, 38]. According to the atlas, the total AP length of the SN that overlaps with the CN III rootlets is 2.25 mm (−10.80 mm to −13.05 mm AP from bregma), nearly 30% of the whole SN (2.25/8 = 28.13%), and, according to the criterion, the optimal sections for staining were approximately 20 coronal sections (80 sections × 28.55% = 22 sections), ranging from sections 11 to 30 out of 80.

#### Staining for Pathological Hallmarks of PD

The 20 optimal sections of SNs were divided into 2 sets for TH and PSer129αS immunostaining. One of every 2 sections among the 20 was picked out to form a set of 10 sections.

For TH staining, a standard protocol was used [32, 38]: SN sections were treated with 3% H_2_O_2_ (5 min; Maixin, Fuzhou, China), 3% Triton X-100 (4 min; Solarbio, Beijing, China), and goat serum (15 min; Maixin, Fuzhou, China) and then incubated with rabbit anti-TH antibody (1:1000, AB152; Millipore, USA) overnight at 4 °C. The sections were then incubated with secondary antibodies (PV-9000, 30 min; ZSGB-BIO, Beijing, China) at 37 °C. Afterwards, the sections were reacted with DAB (DAB-1031Kit, 20×; Maixin, Fuzhou, China) and counterstained with hematoxylin.

For PSer129αS staining, we used a previously-published protocol [30, 41, 42]. The sections were treated with 50 μg/mL proteinase K (Aladdin, Shanghai, China) in 10 mmol/L Tris-HCl, pH 7.8, 100 mmol/L NaCl, and 0.1% Tween-20 (Bio-Rad, USA) at 37 °C for 30 min. After that, sections were treated with 3% H_2_O_2_ (5 min; Maixin, Fuzhou, China), 3% Triton X-100 (4 min; Solarbio, Beijing, China), 10% goat serum (15 min; Maixin, Fuzhou, China) and incubated with a commonly-used rabbit anti-PSer129αS polyclonal antibody (1:100; ab59264, Abcam, UK) [30, 42] overnight at 4°C. On the second day, the sections were incubated with anti-rabbit/mouse secondary antibodies (PV-9000, ZSGB-BIO, Beijing, China) for 30 min at 37 °C. Then, the sections were incubated with DAB (20×; DAB-1031 Kit, Maixin, Fuzhou, China) for 2 min and counterstained with hematoxylin for 1 min.

After immunostaining, all sections were dehydrated in an alcohol gradient and cleared with xylene. Neutral balsam was used to cover the slides and digital images were captured on a CX41 microscope (DP25 camera, CellSens Entry 1.4.1 software; Olympus, Japan). Ten sections containing both the left and right SN regions of each monkey were selected for the counting of TH-positive cells and PSer129αS aggregates.

#### Staining for Gene-Editing Effect and Glial Cell Activation Study

In the immunofluorescence staining, the brain sections from monkey Middle age 2 were washed for 5 min in 0.01 mol/L PBS containing 5% bovine serum albumin (BSA) and 0.3% Triton X-100, and then incubated with a set of primary antibodies (in 0.01 mol/L PBS with 1% BSA and 0.3% Triton X-100) overnight at 4 °C.

In gene-editing effect tests, two sets of primary antibodies were used: (1) anti-EGFP (ab6673, Abcam), anti-NeuN (ABN78, Millipore), anti-Paris (75-195, NeuroMab); and (2) anti-EGFP (ab13970, Abcam), anti-NeuN (MABN377, Millipore), and anti-DJ-1 (ab201147, Abcam).

For glial cell co-staining, another set of primary antibodies was used: anti-GFAP (SMI-21R, Covance Research Products Inc.), anti-Iba1 (019-19741, Wako), and anti-EGFP (ab13970, Abcam).

All the sections were subsequently treated with the corresponding secondary antibodies (1:1000, Alexa-Fluor-conjugated, Invitrogen). DAPI was used to label nuclei and the sections were mounted with 75% glycerol for microscopy.

#### Stereological (Cell) Counting for Pathological Hallmarks of PD

After immunostaining, stereological counting was carried out to quantify the pathological changes. The protocols were adopted from previous studies [38, 40, 43, 44]. A typical immunostained section was screened under a 4× objective (3.5 mm × 2.6 mm), a 20× objective (690.8 μm × 518.1 μm), and a 40× objective (345.4 μm × 259 μm) to locate the SNpc for cell counting, because the clear morphology of nigral dopaminergic neurons are observed under 40× objective [36]. The location of the counting frame on a section of the control side or the gene-edited side was in the same part of the SNpc, in the middle of its long axis. This location was determined by both the anatomical characteristics of the SNpc (ellipsoid and defined ventrally by the cerebral peduncle and medially by CN III) and the cellular properties of the SNpc: pigmented, spindle-shaped cells evenly distribution in the area.

The number of nigral dopaminergic neurons (TH^+^ cells) in the area of the counting frame was counted using ImageJ software (National Institutes of Health, Bethesda, USA) under a 40× objective to ensure correct recognition of the morphology of an individual cell. The averaged density of cells (subject/mm^2^) on the control side and the gene-edited side were statistically analyzed. We found the average density of nigral dopaminergic neurons on the gene-edited side was close to that of PD patients [45].

To verify the reliability of the counting method, the total number of dopaminergic neurons in the SNpc was estimated by the classic method used by Garcia *et al*. [46] and Gundersen *et al*. [40]. The average total number of neurons in the SNpc on the control side was ~14,800, consistent with previous studies (~14,500) [38, 39], suggesting that the counting was reliable. Stereological counting of the nigral PSer129αS aggregates was carried out in the same way.

#### Confocal Imaging and Data Quantification for Gene-Editing Effect and Glial Activation Study

To illustrate the gene-editing effects and glial cell activation, confocal z-stack images were acquired by a Nikon A1 confocal laser microscope system. NIH ImageJ software was used to analyze the fluorescence intensity of immuno-reactive cells in the brain sections from monkey Middle age 2. The fluorescence intensity of Paris immunostaining in EGFP^+^/NeuN^+^ and EGFP^−^/NeuN^+^ nigral cells was analyzed to quantify the expression level of PINK1 and the fluorescence intensity of DJ-1 immunostaining from EGFP^+^/NeuN^+^ and EGFP^−^/NeuN^+^ nigral cells was also analyzed to quantify the expression level of DJ-1. The positive signals of astrocyte immunostaining combined with EGFP^+^/GFAP^+^ or EGFP^−^/GFAP^+^ in the SN area on the gene-edited side were analyzed to quantify the number of astrocytes, and the positive signals of microglial immunostaining combined with EGFP^+^/Iba1^+^ or EGFP^−^/Iba1^+^ in the SN area on the gene-edited side were also analyzed to quantify the number of microglia.

### Statistical Analysis

Statistical analysis was performed with SPSS software (IBM SPSS Statistics 25, free version). Regression was performed with SPSS 25 (regression-curve fit). ANOVA (homogeneous variance), or NPar tests: Mann-Whitney test (non-homogeneous variance) was used to compare the means/distributions of the PD score of each item before (baseline) and after viral injection (last test), latency, accuracy, rotations, TH^+^ cell count, PSer129αS aggregates, Iba1^+^ cell count, GFAP^+^ cell count, and fluorescence intensity of Paris in EGFP-positive or EGFP-negative neurons, or fluorescence intensity of DJ-1 in EGFP-positive or EGFP-negative neurons. Significance was represented as *P <0.05, **P <0.01, ***P < 0.005, ****P < 0.001.

## Results

### Mutation Efficiency Tests

We first designed two sgRNAs targeting the rhesus monkeys’ *PINK1/PARK6* gene (sgRNA-*PINK1*-A and sgRNA-*PINK1*-B) and other two sgRNAs targeting the rhesus monkeys’ *DJ-1/PARK7* gene (sgRNA-*DJ-1*-A and sgRNA-*DJ-1*-B) (Fig. 1A–D), and then tested the efficiency of the sgRNAs in the COS7 cell line from the African green monkey. After transfection of the SaCas9 and sgRNA vectors into COS7 cells, we used polymerase chain reaction (PCR) amplification to determine whether CRISPR/Cas9 induced mutations in the sgRNA-targeted regions. We found that all the sgRNA-*PINK1*-A, sgRNA-*PINK1*-B, sgRNA-*DJ-1*-A, and sgRNA-*DJ-1*-B effectively caused missense mutations in the protein coding regions of the COS7 cells (Fig. S2). To co-edit both the *PINK1/PARK6* and *DJ-1/PARK7* genes, we co-injected the four AAV9s (AAV9-Syn-SaCas9-sgRNA-*PINK1*-A/B, and AAV9-Syn-SaCas9-sgRNA-*DJ-1*-A/B) together into the SNs on one side of the monkeys’ brains, along with an AAV9-Seen-EGFP to label the injection sites.

### Parkinsonian Symptom Assessment

To minimize the influence of individual variations, we used a self-controlled design in which four adult male rhesus monkeys were involved in developing a hemi-Parkinsonism model (Table 1). As age is an important risk factor in the pathogenesis of PD [47], the four monkeys were divided into two age groups: an old group (Old 1 and Old 2: 21.5 ± 2.12 years old) and a middle-aged group (Middle age 1 and Middle age 2: 10 ± 0 years old).

**Fig. 2:**
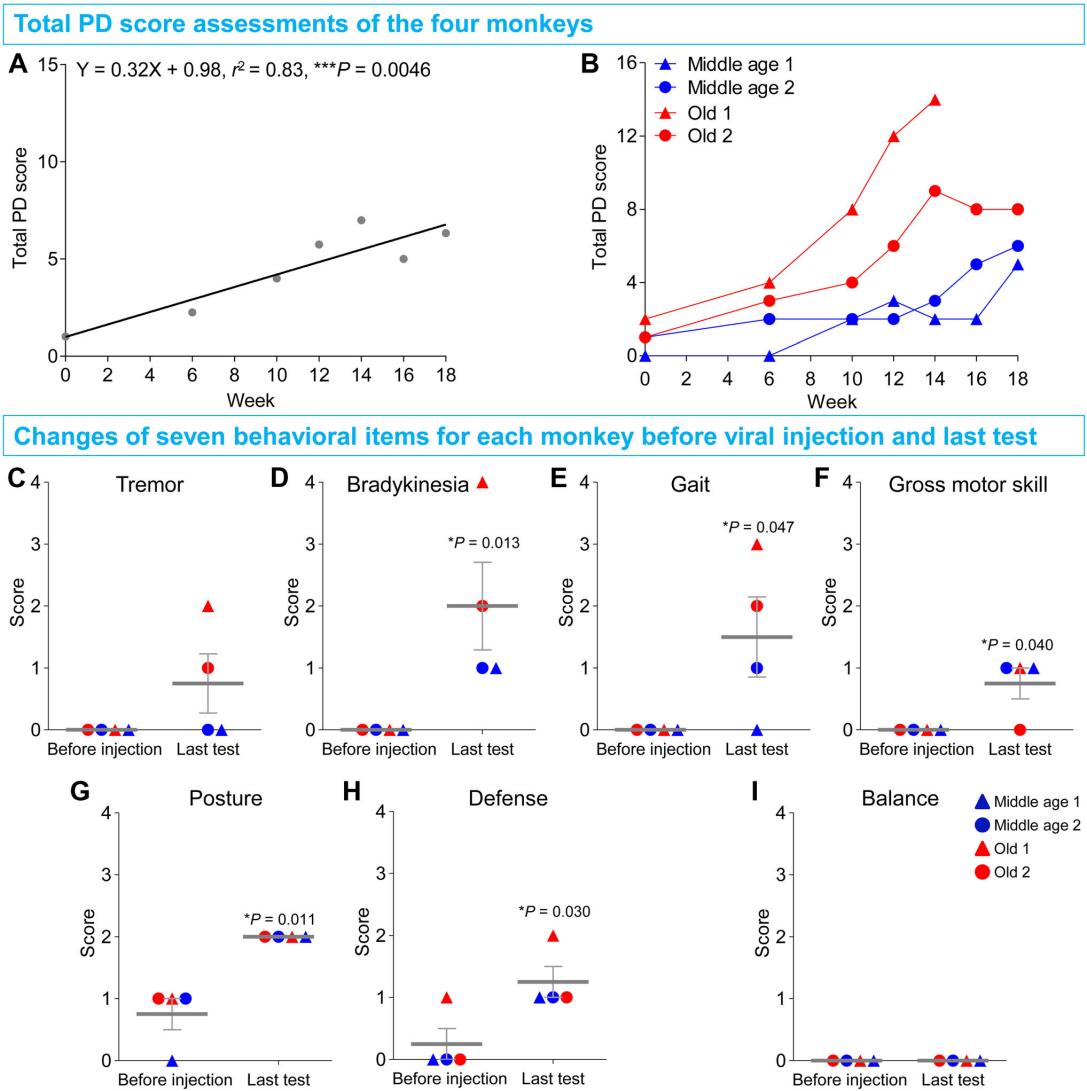
Classic Parkinsonian symptoms induced by the AAV9-delivered CRISPR/Cas9 editing of *PINK1* and *DJ-1* genes in the monkey SN. **A** Averaged total PD scores of the four monkeys assessed by the improved Kurlan scale progressively increase after *PINK1* and *DJ-1* gene-editing, and the correlation between the total PD score and time (Week) is significant. **B** The total PD score of each monkey also increases with time (Week), and the scores of the old monkeys are always higher than those of the middle-aged monkeys. **C–I** Changes of the seven behavioral items of the improved Kurlan scale for each monkey between baseline (before viral injection) and the last test [data from monkey Old 1 are from week 14 (sacrificed early due to poor condition), while the data of the others are from week 18]. **C–F** Tremor, bradykinesia, gait, and gross motor skill all increase from a baseline of zero. The scores of the old monkeys are usually higher than those of the middle-aged monkeys. **G, H** Abnormal posture and defense abnormality increase significantly compared with baseline. **I** Loss of balance, which usually only appears at the late stage of monkey PD, does not appear. Data in **C–I** are presented as the mean ± SEM.

The first step in creating the model was to deliver the AAV9-mediated CRISPR/Cas9 gene editing systems into the SN on one side in each subject. To ensure coverage of the whole targeted SNs, we used the procedure developed previously in our lab [26, 31] (Fig. 1E–G, Table 2). The SN on the other side served as the control, into which AAV9-SaCas9-SgControl was injected. Six weeks after the viral injections, the total PD score of improved Kurlan scale for each monkey was recorded. The average total PD scores of all the gene-edited monkeys progressively increased over time (Fig. 2A). The total PD score curve for each monkey over time is shown in Fig. 2B. The end-points of the two old monkeys were markedly higher than those of the middle-aged monkeys. The results revealed that the two old monkeys eventually developed severe and moderate stages of PD with total PD scores of 14 and 8 respectively; PD progression of the old monkeys was much faster and more severe than their middle-aged counterparts, which just developed mild stage of PD with total PD scores of 5 and 6, respectively.

The next step was a close check on the changes in the scores for the seven behavioral items that make up the improved Kurlan scale from the beginning to the end of the experiment. The items (tremor, bradykinesia, gait, defense, posture, balance, and gross motor skills) were all zero or very low at the commencement of the experiment, had all appeared or increased by the end of the experiment, except for “balance”, which is not usually observed unless a monkey is at a very late stage of PD [34, 48] (Fig. 2C–I). According to the UK Parkinson’s Disease Society Brain Bank clinical diagnostic criteria [3], the core clinical symptoms in PD patients include the essential criterion of bradykinesia, and at least one of the following: resting tremor or postural instability (reflected as gait and gross motor skill disabilities, Table S1). Therefore, the above data almost perfectly matched the diagnostic criteria of PD in humans (Movies S1 and S2). That is, the gene-edited monkeys were adult monkey models with Parkinsonism. Again, like the total PD scores, the scores for the individual items in the old group were generally higher than those in the middle-aged group (Fig. 2C–E), indicating their greater sensitivity to the gene-editing procedure.

**Fig. 3:**
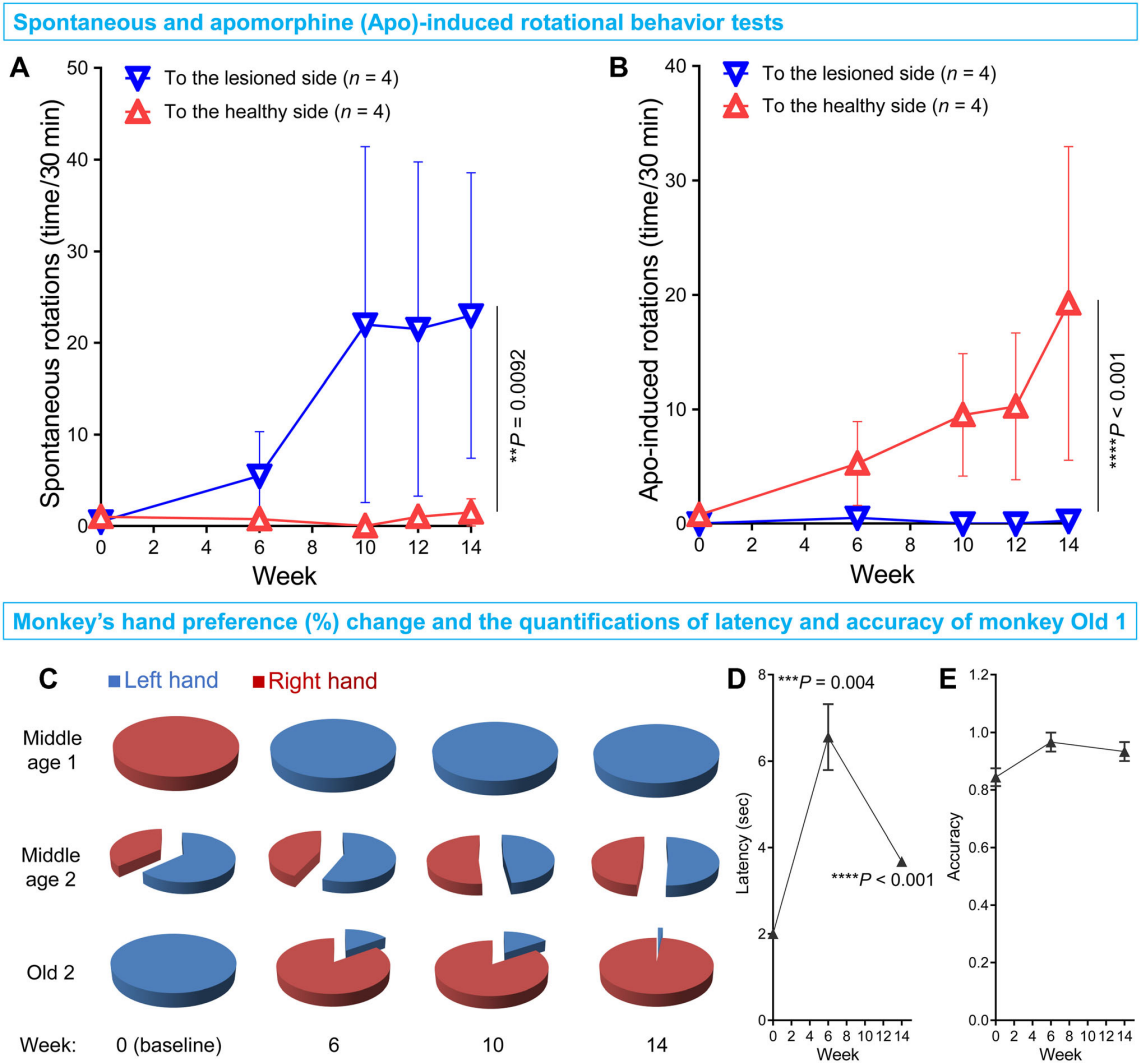
Quantification of functional deficits in the nigrostriatal dopaminergic system by *PINK1* and *DJ-1* gene-editing in the adult monkey SN using the apomorphine (Apo)-induced rotational behavior test, and quantification of the fine motor skill deficits caused by the *PINK1* and *DJ-1* gene-editing using a food-grasping task. **A** Spontaneous rotational behavior to the lesioned side progresses after gene-editing. **B** Apo-induced rotational behavior progressively reverses to the healthy side after gene-editing. **C** The hand preference changes after the viral injections, except for monkey Old 1. **D** Monkey Old 1 displays a significant increase of latency in the food-grasping task after gene-editing. **E** Accuracy of monkey Old 1 does not change from baseline. Data are presented as the mean ± SEM.

Following above classical PD symptom assessment, a pharmacological test of dopaminergic system function was used and measured by the rotation test paradigm. In the test, the monkey is given Apo, an agonist of the dopamine D1 receptor. As the postsynaptic dopaminergic receptors in the striatum of the lesioned side are more sensitive to Apo than those in the healthy side, this imbalance causes the monkey to rotate to the healthy side after Apo administration [49, 50]. After the viral injections but prior to the administration of Apo, the monkeys slowly developed a tendency to rotate to the lesioned side due to the damage to the dopaminergic system by the gene-editing (Fig. 3A). After administration of Apo, the rotation was reversed to the healthy side (Fig. 3B). These data demonstrated a significant impairment of the PD-related dopaminergic system in the monkey brain after gene-editing (Movie S3).

Another important behavioral dysfunction in PD patients is deficits of fine movement, which we tested in monkeys using a food-grasping task. To prepare for this test, the side of SN lesioning was selected based on the hand preference of each monkey: Middle age 1 and Old 1 were 100% right-handed, so the AAV9s-Cas9 targeting the *PINK1* and *DJ-1* genes was injected into their left SNs. Conversely, monkeys Middle age 2 (63.33% left-handed) and Old 2 (100% left-handed) were mostly left-handed, and thus the genes in their right SNs were edited. After the viral injections, three of the four monkeys switched their preferred hand in the food-grasping task (Fig. 3C), clearly demonstrating a fine movement deficit in their preferred hand because they were not able to use their previously preferred hand to pick up food effectively and had to switch to the other hand (Movie S4). The latencies for three of the monkeys were increased (Fig. S3A) and accompanied by decreased accuracy (Fig. S3B). Only Old 1 maintained its hand preference (data not shown), but with a significantly increased latency (Fig. 3D) and unchanged accuracy (Fig. 3E), revealing less severe fine movement deficits.

In summary, co-editing *PINK1* and *DJ-1* genes by AAV9s-delivered CRISPR/Cas9 systems in the SNs of adult monkeys was effective in eliciting typical PD symptoms. The next step was to investigate whether it can cause the pathological hallmarks of PD.

### Assessment of Pathological Hallmark

Three monkeys were sacrificed for the study of PD pathology. The brains of monkeys Middle age 1 (10 years old) and Old 1 (23 years old) were used for classical PD pathology validation, while the brain of monkey Middle age 2 (10 years old) was used for viral transfection and gene-editing effect tests.

**Fig. 4:**
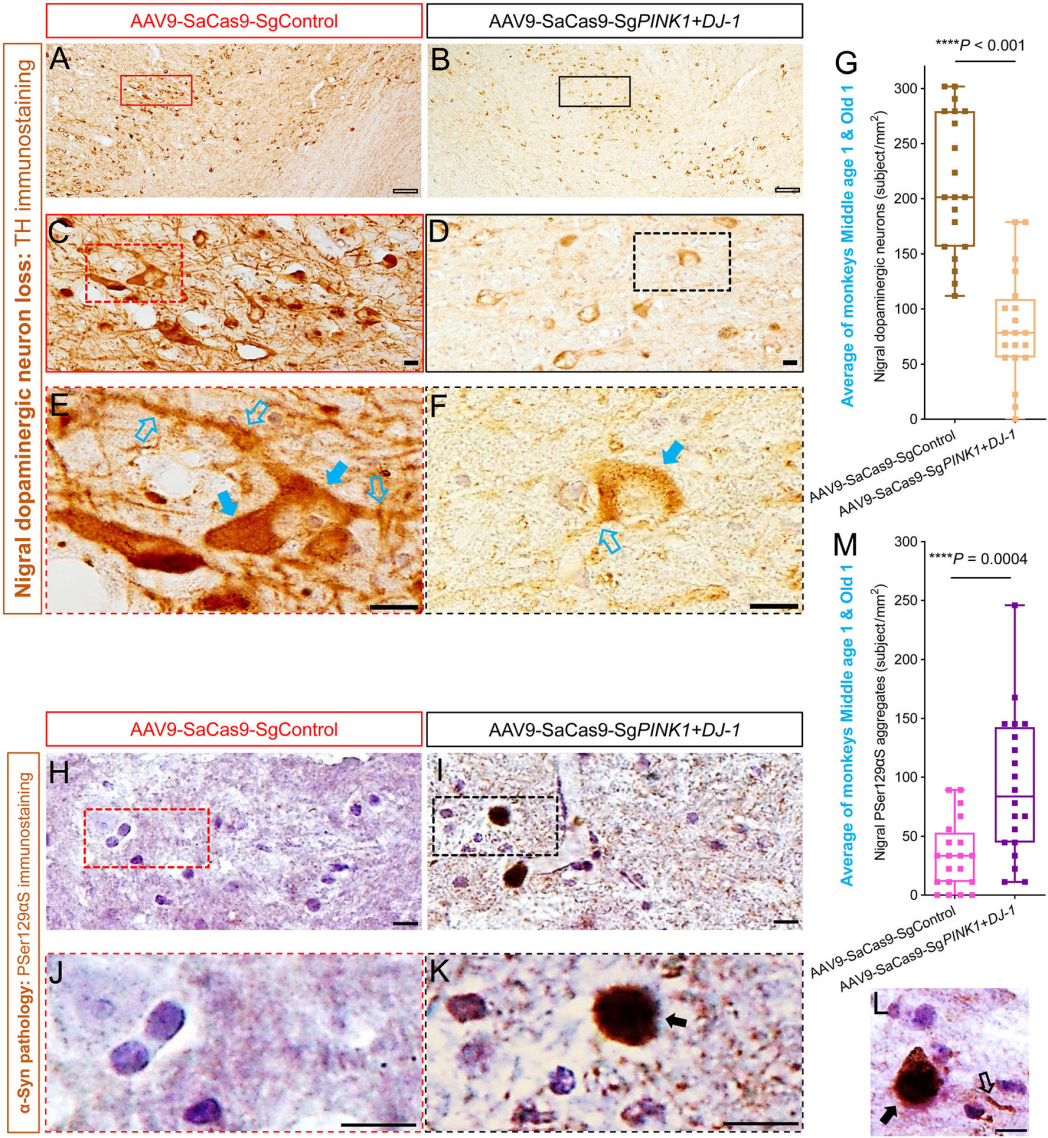
Pathological hallmarks of PD in the SN region of *PINK1* and *DJ-1* gene-edited monkey brain. **A, B** TH immunostaining images of the AAV9-SaCas9-SgControl SN (**A**) and the AAV9-SaCas9-Sg*PINK1*+*DJ-1* SN (**B**) of monkey Old 1 at low resolution (4×). The image in **B** appears pale and weak with fewer surviving neurons compared with **A**. **C, D** Moderate resolution (20×) images of boxed areas in **A** and **B**. Typical dopaminergic neurons are evident in **C**, and many deformed dopaminergic neurons, with weakly-stained somata and almost completely lost neurites are shown in **D**. **E, F** High resolution (40×) images of the boxed areas in **C** and **D**. A typical dopaminergic neuron with intact soma (solid blue arrows) and branched processes (hollow blue arrows) can be seen in **E**, and a very weakly-stained dopaminergic neuron with incomplete soma boundary (solid blue arrow) and a residual process (hollow blue arrow) is shown in **F**. **G** Over 61% of nigral dopaminergic neurons are lost in AAV9-SaCas9-Sg*PINK1*+*DJ-1* on the edited side compared with the AAV9-SaCas9-SgControl side (an average of monkeys Old 1 and Middle age 1). **H, I** PSer129αS immunostaining images under high resolution (40×) of the AAV9-SaCas9-SgControl SN (**H**) and the AAV9-SaCas9-Sg*PINK1*+*DJ-1* SN (**I)** from monkey Old 1. Clear PSer129αS aggregates can be seen in **I**. **J, K** Enlarged images of the defined areas in **H** and **I** showing stained nuclei in **H** and a typical darkly-stained PSer129αS aggregate in **I** (arrow). **L** Typical example of a PSer129αS aggregate (solid arrow) and a PSer129αS deposit in a neuronal process (hollow arrow) in the AAV9-SaCas9-Sg*PINK1*+*DJ-1* SN of monkey Old 1. **M** Numbers of PSer129αS aggregates in the AAV9-SaCas9-Sg*PINK1*+*DJ-1* and the AAV9-SaCas9-SgControl SNs (average of monkeys Old 1 and Middle age 1). Scale bars: hollow black, 100 μm; solid black, 10 μm. Data in **G** and **M** are the median with minimum and maximum (*n =* 20 sections).

The most important pathological hallmark of PD is the severe loss of dopaminergic neurons and marked morphological changes of the surviving dopaminergic neurons in the SNpc [3, 4]. TH immunostaining, which is a classic method of visualizing dopaminergic neurons, showed that the SNs on the genetically-edited side became pale and blurred in lower resolution images (4×, Fig. 4A, B), and the surviving dopaminergic neurons were severely deformed compared with the those on the AAV9-SaCas9-SgControl side in higher resolution images (20×, Fig. 4C, D). Further closer observation showed that the somata of the dopaminergic neurons on the genetically-edited side were blurred and the neurites were almost lost (40×, Fig. 4E, F) compared with those on the control side. Cell counts revealed that >61% of the dopaminergic neurons (average of the two monkeys) were lost on the genetically-edited side compared with those on the AAV9-SaCas9-SgControl side (Fig. 4G). In the two individuals, the dopaminergic neuron loss was approximately 64% in Old 1 and approximately 58% in Middle age 1. Importantly, dopaminergic neurons on the AAV9-SaCas9-SgControl side of the two monkeys were not significantly different from those in the two age-matched normal control monkeys used in our previous studies [26, 30] either in morphology or cell number (Fig. S4), indicating that the AAV9 injections and the AAV9-SaCas9-SgControl agent did not cause any marked lesions in the SNs. Taken together, the AAV9-mediated CRISPR/Cas9 editing of the two genes successfully induced the most important pathological hallmarks of PD: severe loss of dopaminergic neurons and clear morphological changes of the surviving dopaminergic neurons.

Another important PD hallmark, Lewy body pathology is commonly illustrated by phosphorylated-serine129 α-synuclein (PSer129αS) immunostaining. Although classical Lewy bodies were not identified in this study, α-synuclein pathology (also termed PSer129αS aggregates), was clearly revealed by the staining. α-Synuclein pathology can be considered an early stage of Lewy pathology for the following reasons: (1) aggregated α-synuclein is the major component of the Lewy body [51, 52], and ~90% of the α-synuclein aggregates consist of PSer129αS [53]; and (2) Lewy bodies are probably triggered by abnormal accumulation of PSer129αS in the cytoplasm [54]. Identified by antibody ab59264, one of the most commonly used antibodies for PSer129αS immunostaining [42, 55], typical PSer129αS aggregates were found in the cytoplasm of the nigral neurons on the genetically-edited side in comparison to that of the control side (Fig. 4H, I). Enlarged images showed the morphological details of the PSer129αS aggregates (Fig. 4K), which were also identified in cell processes (Fig. 4L). But these aggregates were not observed on the control side (Fig. 4H, J). Quantitative analysis revealed that the number of PSer129αS aggregates in the SN on the genetically-edited side was more than double that on the control side (average of the two monkeys) (Fig. 4M).

In summary, these results strongly suggested that the key pathological hallmarks of PD had been successfully induced by co-editing *PINK1* and *DJ-1* genes *via* AAV9-delivered CRISPR/Cas9 systems in adult monkey brains. Together with the classic symptoms demonstrated above, we propose that the gene-editing of PD monkeys has been successfully developed.

### Consequences of Viral Transfection and Gene Editing

**Fig. 5:**
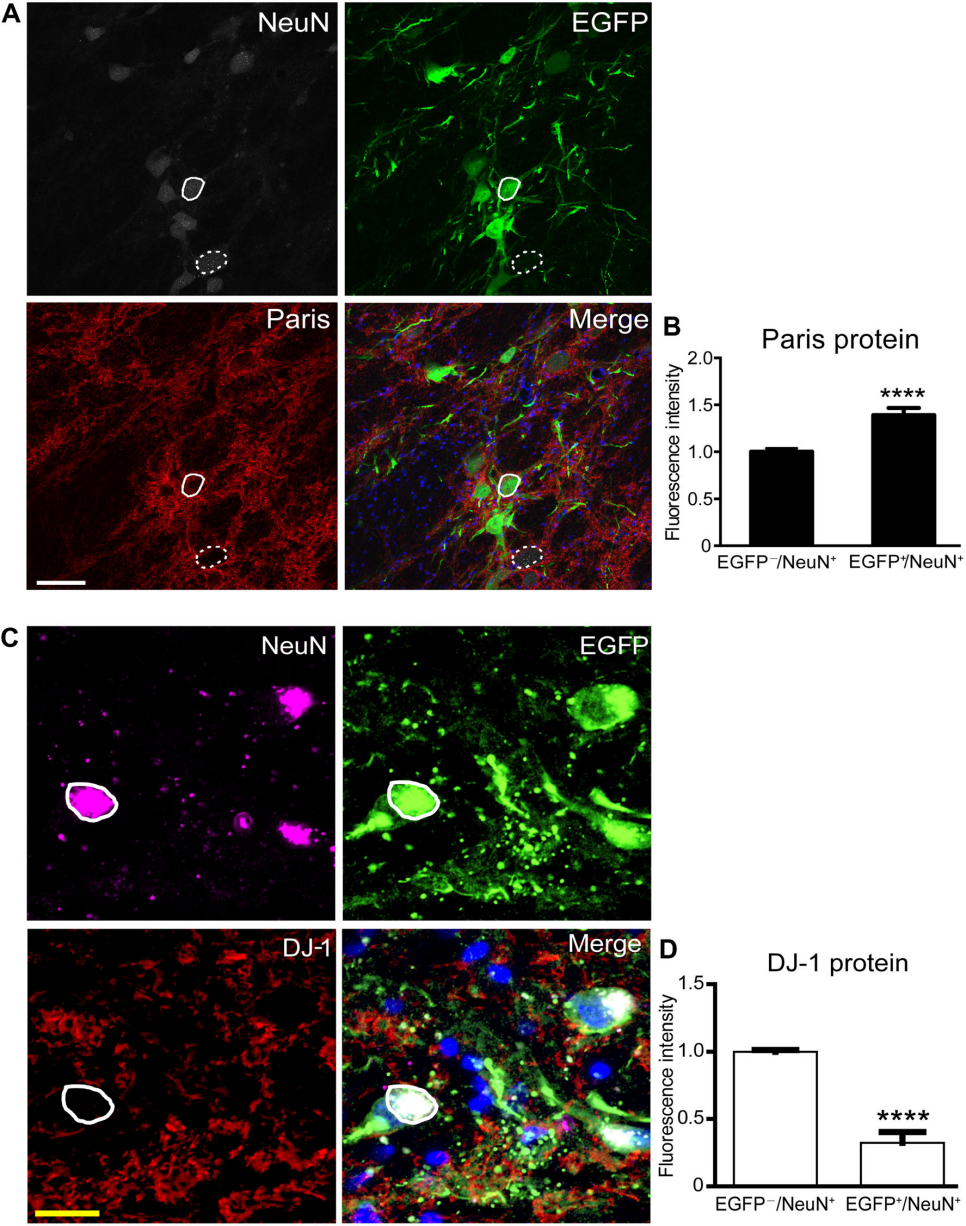
Immunofluorescence images of up-regulated Paris for indicating *PINK1* down-regulation, and the reduced *DJ-1* expression level. **A, B** Fluorescence images of Paris (**A**, red) and averaged intensity (**B**) in EGFP-negative and EGFP-positive neurons (NeuN^+^, gray) (EGFP-negative neurons, *n =* 18; EGFP-positive neurons, *n =* 30; ****P <0.001). **C, D** Fluorescence images of DJ-1 (**C**, red) and averaged intensity (**D**) in EGFP-negative or EGFP-positive neurons (NeuN^+^, purple) (EGFP-negative neurons, *n =* 24; EGFP-positive neurons, *n =* 24; ****P <0.001). Scale bars: white, 100 μm; yellow, 20 μm. Data are presented as the mean ± SEM.

To demonstrate that the gene-editing is effective *in situ*, we found that the expression of Paris/ZNF746, a critical substrate of the PINK1/Parkin signal pathway, was significantly increased in the SN region of the AAV9-CRISPR/Cas9 genetically-edited monkey Middle age 2 (Fig. 5A), as measured by the Paris level in EGFP-positive neurons compared with adjacent EGFP-negative neurons (Fig. 5B). These results not only revealed that the AAV9s successfully transfected the nigral neurons, but also caused functional loss of the PINK1/Parkin pathway, leading to the up-regulation of Paris, which was consistent with that in the human PD brain [56]. Meanwhile, we tested the expression of DJ-1 protein in EGFP-positive neurons compared with adjacent EGFP-negative neurons in the gene-edited SN, and found a significant down-regulation of DJ-1 *in situ* (Fig. 5C, D), which may also contribute to PD pathogenesis. Collectively, loss-of-function mutations in the *PINK1* and *DJ-1* genes were induced by the nigral AAV9-delivered CRISPR/Cas9 gene-editing procedure and both together probably led to PD pathogenesis.

## Discussion

In this study, we used an AAV9-delivered CRISPR/Cas9 gene-editing system to co-edit the *PINK1* and *DJ-1* genes in the unilateral SN of the adult rhesus monkey brain to investigate the possible causal relationship between these mutations and PD development. After the procedure, we observed that nearly all the clinical PD symptoms, such as tremor, bradykinesia, gait abnormality, and dramatic hand preference changes, as well as the rotational behavior induced by Apo administration. All the important pathological hallmarks of PD were induced as well, including a loss of >61% of the dopaminergic neurons in the gene-edited SNs, and the appearance of phosphorylated α-synuclein aggregations (an early stage of Lewy pathology) in the SNs. Together, these results revealed that all the classical PD phenotypes were induced by the AAV9-delivered CRISPR/Cas9 system, and strongly suggested that a new gene-edited PD monkey model has been successfully developed. In addition, we observed age-dependent severity in Parkinsonian symptoms and pathological changes, which suggested that both the genetic and aging factors play important roles in the development of PD.

Previous studies using virus-based transgenic technics in adult non-human primate brains induced some of the pathological PD changes but lacked the core clinical symptoms [13, 14]. To solve these issues, we made two important technical improvements in this study. First, based on our and others’ previous studies, it is known that to elicit PD symptoms, the loss of nigral dopaminergic neurons has to be at least 50% or above in the SN on one or both sides [25, 26]. To achieve this level of cell death, the AAV9s must be delivered to all of one side of the targeted SN. As SN is thin, flat, and located deep within the ventral midbrain, it is difficult to deliver the virus to the whole SN through the normal vertical infusion routes. In this study, unique oblique injection routes (Fig. 1F) were designed based on our previous study [26]. To thoroughly cover the SN on one side, we injected virus along three parallel paths through the SN, with an average of six injection sites along each path. Therefore, an array of 18 viral injection sites was infused in each SN, which ensured complete coverage of the targeted SN (Fig. 1G). To ensure the accuracy of each injection path, we used an accurate MRI-based deep brain structural localization procedure with an error of <1 mm that was developed in our lab [31]. The result was confirmed by the anatomical examination of brain sections obtained after sacrifice (Fig. S5). Second, unlike previous studies that usually manipulated only one gene [17, 57], we edited both the *PINK1* and *DJ-1* genes at the same time. This *“*dual-gene-editing*”* strategy effectively increased the dopaminergic neuron death in the SN. This was supported by our preliminary experiments in which the mutation of either *PINK1* or *DJ-1* alone caused only approximately 40% dopaminergic neuronal loss (Fig. S1), so the monkeys showed no PD symptoms.

On the other hand, studies on genetically-manipulated rodent PD models have shown that no typical motor symptoms of PD occur in either *PINK1*, *parkin*, or *DJ-1* gene-knockout alone [58–60] or knockout of all three genes together [61]. The loss of dopaminergic neurons in the SN was not significant either, and only abnormal dopaminergic release and synaptic plasticity in the striatum were reported [58–61], indicating that the above gene deletions in rodents are not enough to induce the neuropathological and clinical features of PD [61]. In our study, the core phenotypes of PD, which were highly consistent with the phenotypes of human PD patients, were successfully induced by *in situ* gene-editing in the SN of the rhesus monkey. This is the first rhesus monkey model of etiological PD and provides an indispensable platform for the pathogenesis, early diagnosis, and treatment of PD.

As a new endeavor to develop a gene-edited PD monkey model, there are inevitable limitations of the current study. First, the true incidence rate of PD in monkeys caused by gene manipulation is not clear. From the existing data, all 4 of the experimental monkeys showed PD symptoms to different degrees. Therefore, the incidence was 100%, which is already very high. Due to their high value, using 3–4 monkeys in an experiment is a common practice [26, 62, 63]. However, from the perspective of judging the true incidence, more animals are needed, and this will be solved in future experiments. Second, the onset age of early familial PD is approximately 30–50 years old [64], which is the same as that of the middle-aged monkeys in this experiment (10-year-old monkeys are equivalent to 30–40-year-old humans [65]). Our results showed that these middle-aged monkeys also developed PD symptoms, even though to a lesser extent than the older ones. This suggests that aging is an important factor in the development of PD. But the extent to which this model truly mimics the developmental process of early-onset familial PD is still unclear. In follow-up studies, we plan to investigate both the clinical and pathological changes of the model during the course of disease development, and compare this with the clinical results from the early-onset familial PD patients.

In summary, we have successfully developed a gene-editing monkey model with the key PD phenotypes using an AAV9-delivered CRISPR/Cas9 system to co-edit the *PINK1* and *DJ-1* genes in the SN region of the adult monkey brain. In addition, we also observed age-dependent severity in Parkinsonian symptoms, loss of nigral dopaminergic neurons, and α-synuclein pathology, which demonstrated that both genetic and aging factors play important roles in the development of PD. This work paves an important path for the exploration of genetic mechanisms of PD development in non-human primates, provides a new platform for the screening of biomarkers of the early diagnosis and prevention of PD, and a new possibility for the study of interactions between environmental risk factors and genetic mutations in the pathogenesis of PD.

## Supplementary Information

Below is the link to the electronic supplementary material.

Supplementary file1 (PDF 803 KB)

Supplementary file1 (ZIP 19258 KB)
