# Supplementary material for: Co-editing PINK1 and DJ-1 Genes Via Adeno-Associated Virus-Delivered CRISPR/Cas9 System in Adult Monkey Brain Elicits Classical Parkinsonian Phenotype
Source: Neurosci Bull. 2021 Jun 24;37(9):1271–88. doi: 10.1007/s12264-021-00732-6 (PMC8423927; doi:10.1007/s12264-021-00732-6)
Supplement: Supplementary file 1 — Supplementary file1 (PDF 803 KB) [file 12264_2021_732_MOESM1_ESM.pdf]

Supplementary Materials

Supplementary Figures

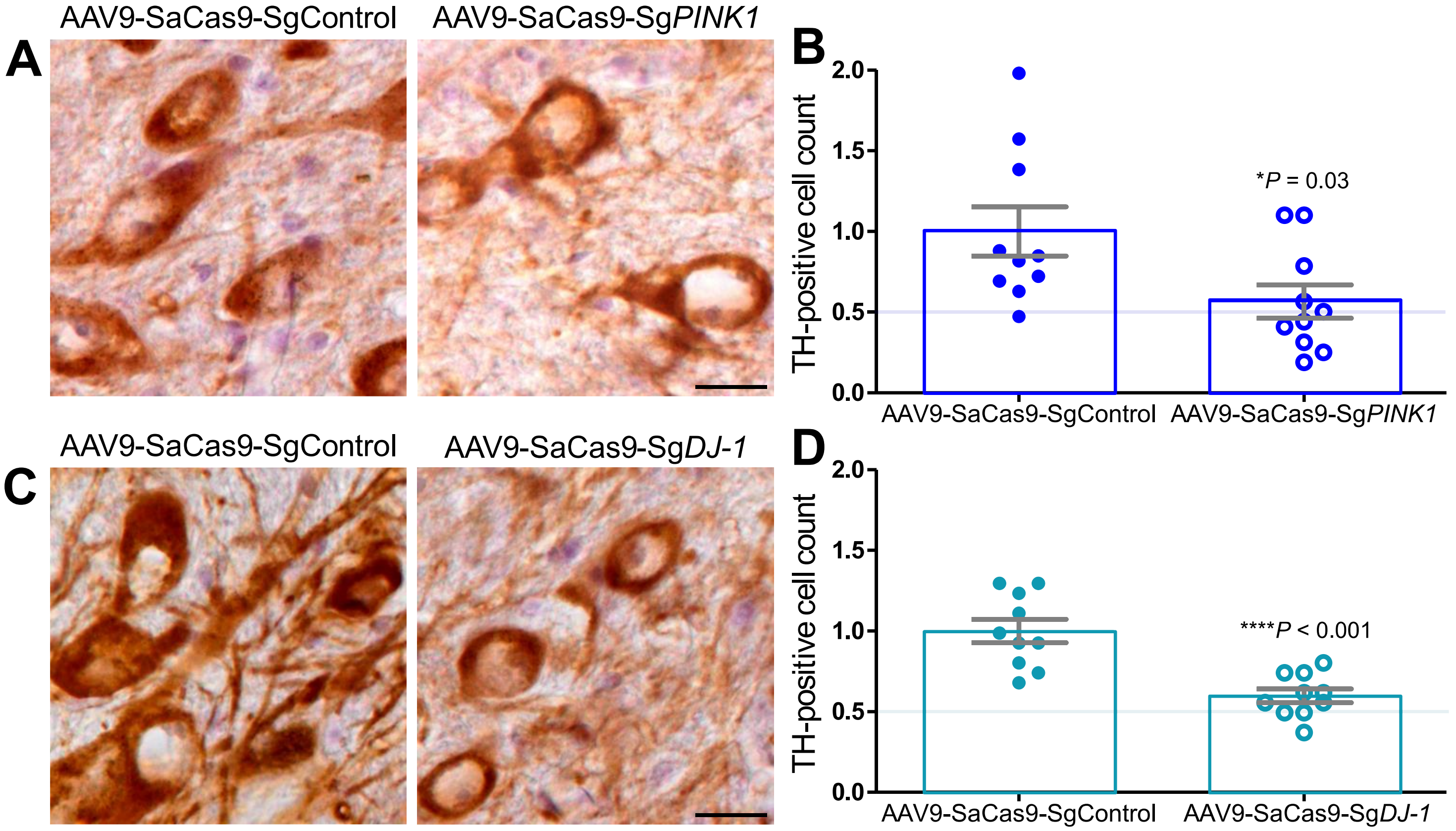

Fig. S1 Nigral dopaminergic cell loss induced by single-gene editing of *PINK1* or *DJ-1* with AAV9-delivered CRISPR/Cas9. **A, B** Representative images (**A**) and counts (**B**) of TH-positive neurons in the SNs on the *PINK1* gene-edited and control sides (scale bar, 20 μm). **C, D** Representative images (**C**) and counts (**D**) of TH-positive neurons in the SNs on the *DJ-1* gene-edited and control sides (scale bar, 20 μm). Data are presented as the mean ± SEM.

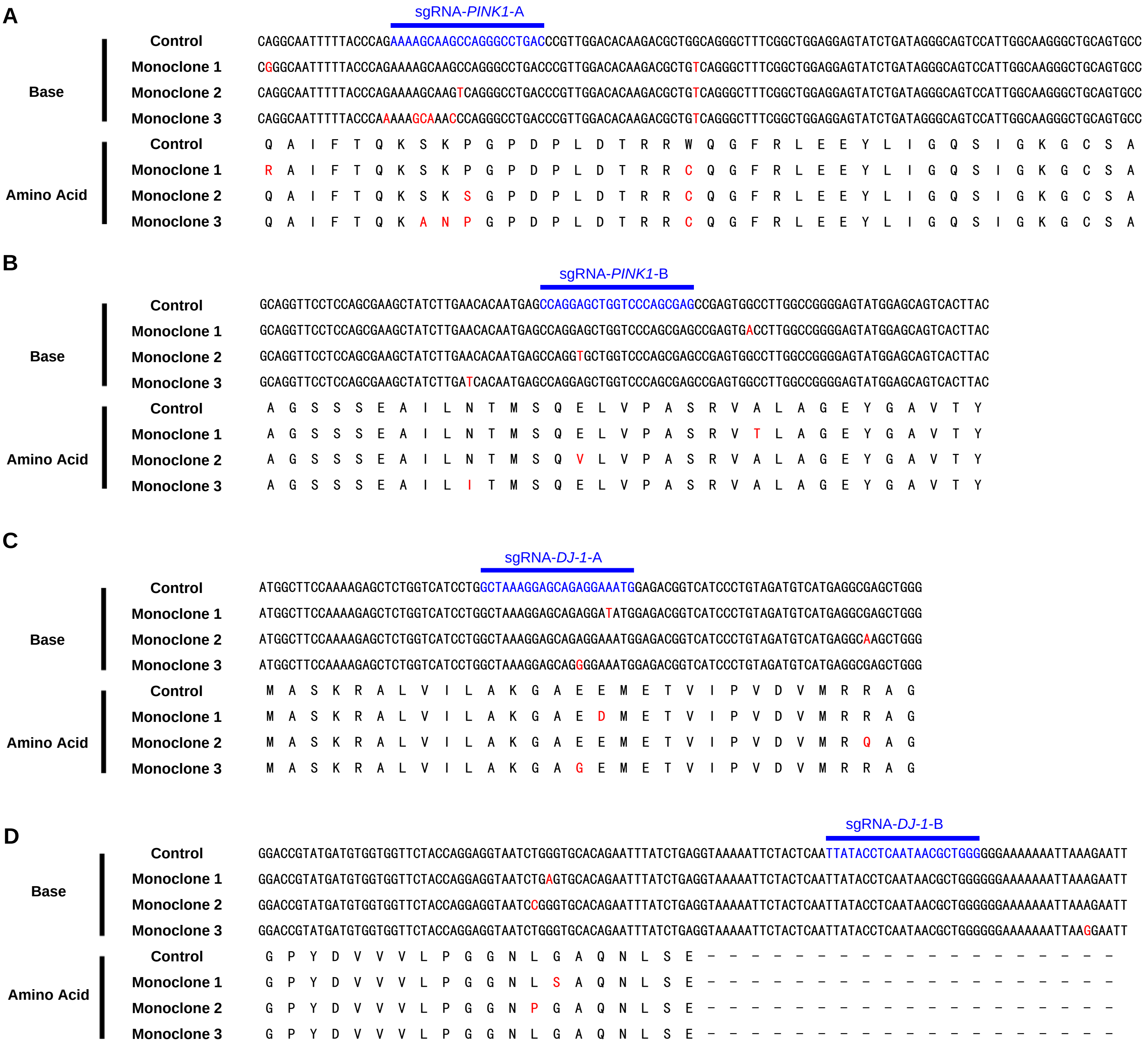

Fig. S2 Identification of the genetic mutations caused by SaCas9 and sgRNAs. **A–D** Examples of monoclone mutations near the target site caused by sgRNAs in COS7 cells (red highlights, mutated bases and amino-acids; blue highlights, sgRNA target sites).

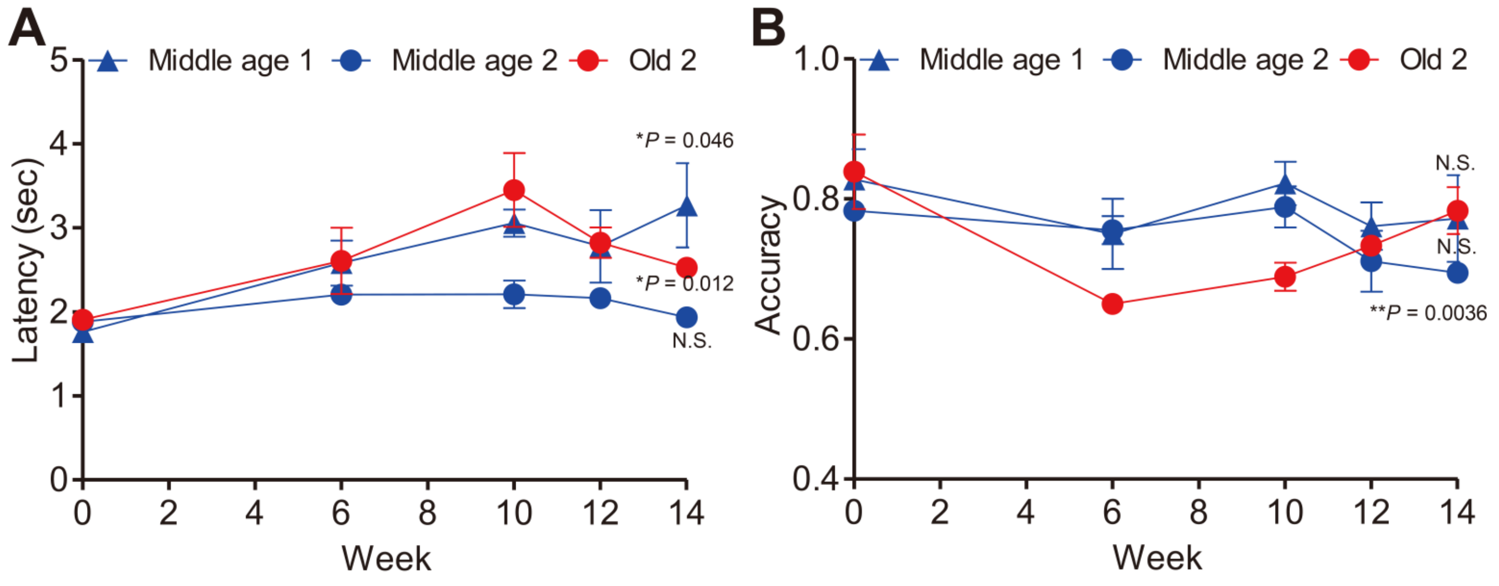

Fig. S3 Changes of latency (**A**) and accuracy (**B**) of the three monkeys that altered their hand preference after AAV9-mediated CRISPR/Cas9 co-editing of the *PINK1* and *DJ-1* genes. Data are presented as the mean ± SEM. N.S., not significant.

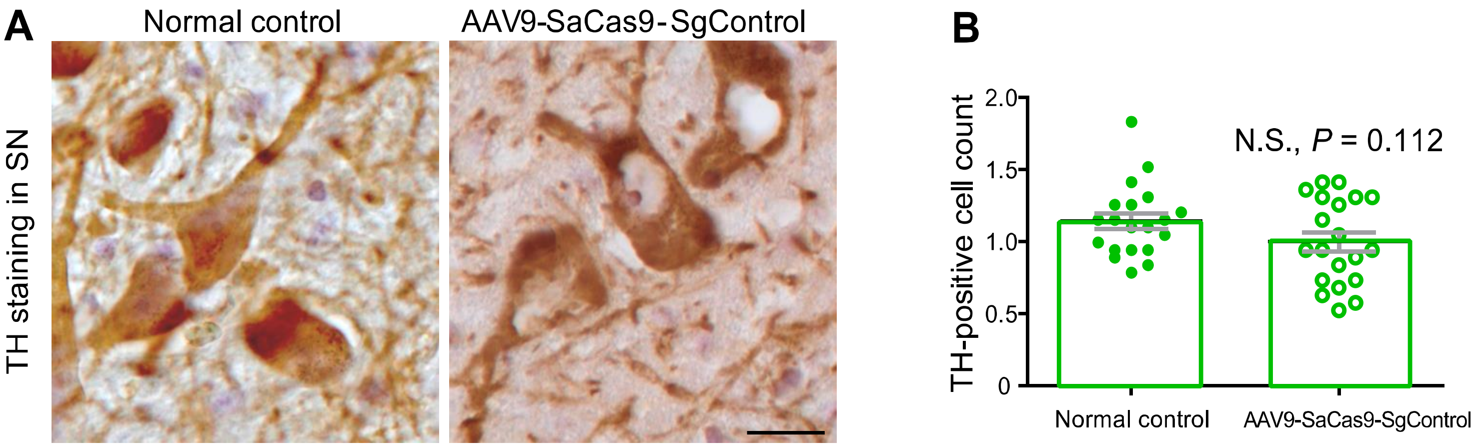

Fig. S4 Representative images (**A**) and numbers (**B**) of TH-immunostained nigral cells in the two age-matched normal control monkeys used in our previous studies and the control sides of the monkeys Old 1 and Middle age 1 in the current study. There are no obvious morphological differences in nigral TH-positive cells between these two groups in **A** (scale bar, 20 μm). Data in **B** are presented as the mean ± SEM.   N.S., not significant.

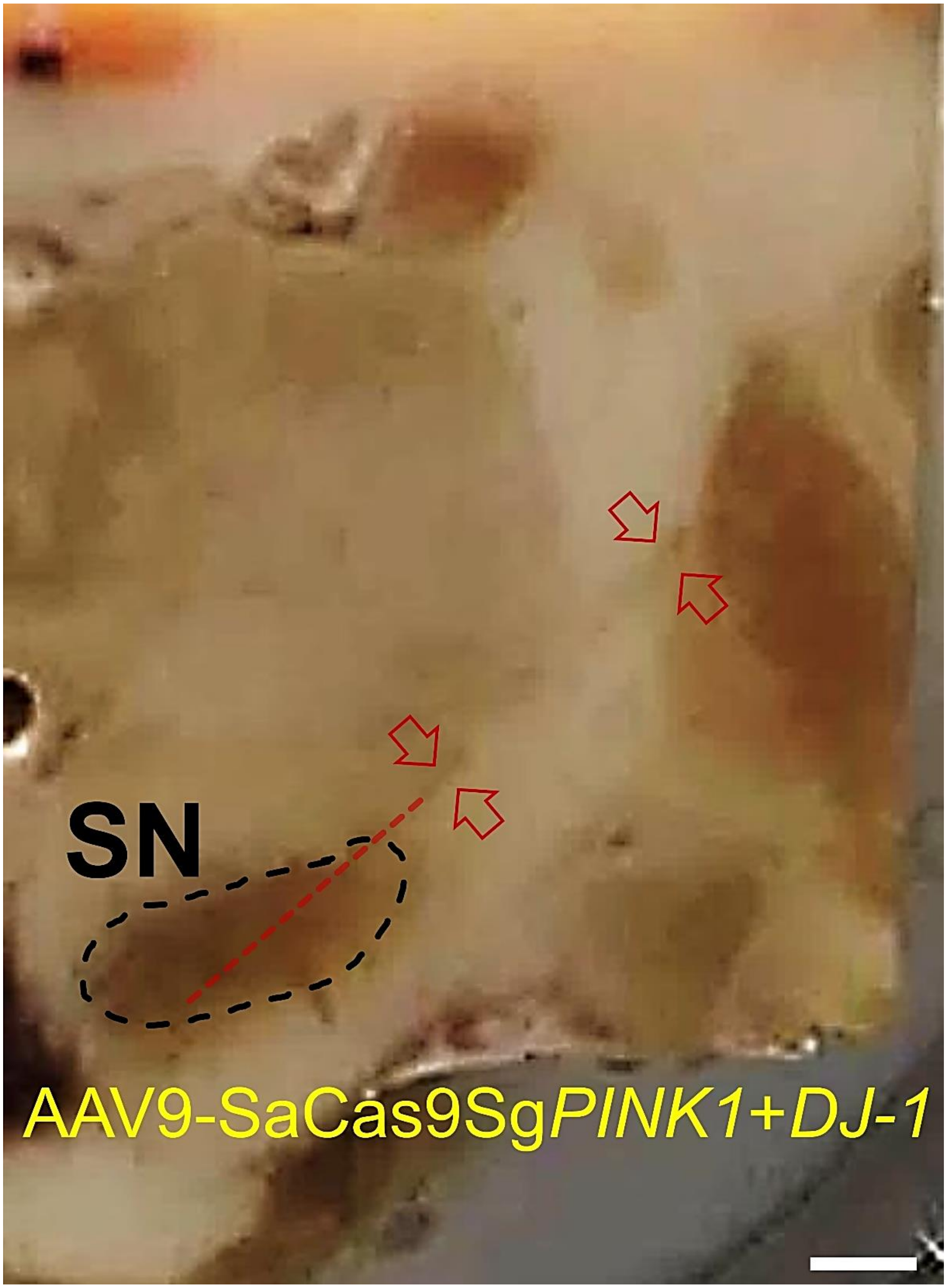

Fig. S5 An accurate injection track of the AAV9-mediated CRISPR/Cas9 gene editing procedure. The red arrows indicate the virus-injection track (red dashed line), which accurately targeted the gene-edited SN region (black dashed line) in brain frozen section of monkey Old 1 (scale bar, 2 mm).

Immunofluorescence Images of Nigral Astrocytes and Microglia with the EGFP Signal in Monkey Middle Age 2

To exclude potential inflammation due to the activation of glial cells by the viral injections, we tested whether the nigral astrocytes and microglia were transfected and activated by the virus by comparing the $EGFP^+$ area (Fig. S6A) with the $EGFP^-$ area in the gene-edited SN region (Fig. S6B). We found that neither the $GFAP^+$ cells nor the $Iba1^+$ cells co-expressed the EGFP signal (Fig. S6A), indicating that nigral glial cells were not transfected by the virus. Moreover, we found no evident morphological changes in nigral glial cells (Fig. S6A, B). Quantitative analysis showed that both the $GFAP^+$ and $Iba1^+$ cell counts in $EGFP^+$ area of the SN region were not significantly higher than that in the $EGFP^-$ area (Fig. S6C, D), suggesting that the amount of glial cells were not increased during the AAV9-mediated gene-editing procedure.

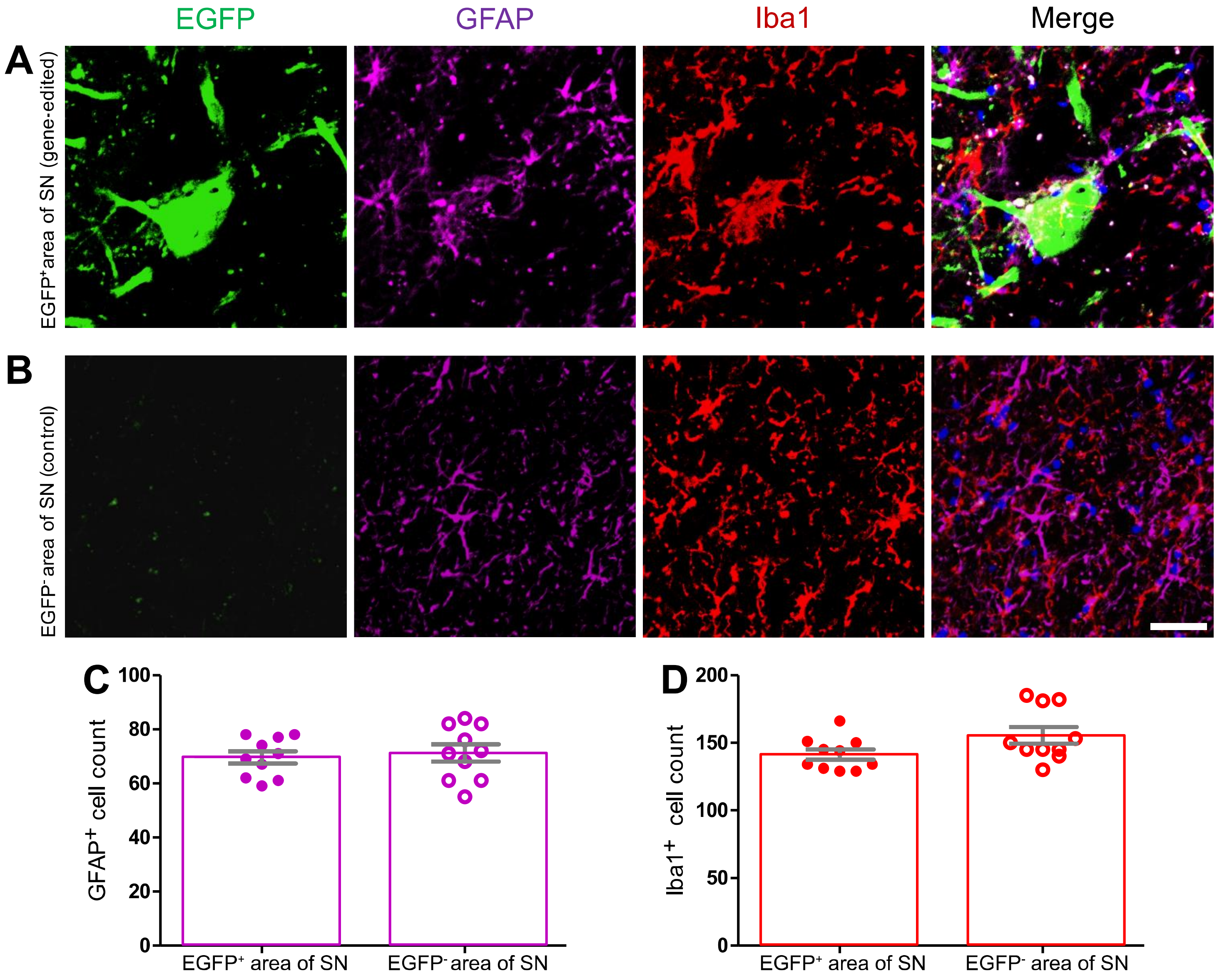

Fig. S6 Immunofluorescence images and quantification of nigral glial cells. **A, B** Astrocytes (GFAP$^+$) and microglia (Iba1$^+$) immunostaining combined with the EGFP$^+$ signal in the gene-edited SN region (**A**) or combined with the EGFP$^-$ signal in gene-edited SN region (control) (**B**) (scale bar, 40 μm). **C, D** No significant changes of the cell counts for astrocytes (GFAP$^+$) in the gene-edited SN region (P = 0.687) (**C**) and microglial cells (Iba1$^+$) in the gene-edited SN region (P = 0.066) (**D**). Data are presented as the mean ± SEM.

Table S1 Improved version of the Kurlan scale (a Monkey-Parkinsonism Rating Scale)

Part A. Parkinsonian features (total score = 20)

1. Tremor (L/R): like the tremor of PD patients

0-absent

1-slight-low amplitude and only intermittently present

2-moderate-moderate amplitude and present most of the time

3-severe-high amplitude, virtually continuous, interferes with function

2. Posture: different from the postural instability of PD patients, not applicable to PD patients

0-normal, erect

1-stooped

2-face down

3. Gait: partly like the postural instability of PD patients

0-normal, use all four limbs smoothly

1-walks slowly

2-markedly impaired, able to ambulate but very slowly and with effort

3-severe decrease in ability to ambulate

4-unable to ambulate

4. Bradykinesia (generalized): like the bradykinesia of PD patients

0-normal speed and facility of movement

1-mild slowing of overall movements

2-moderate slowing of movements

3-severe slowing of movements: slow, labored, and difficult to initiate and maintain movement

4-essentially no movement (akinetic)

5. Balance: partly like the postural instability of PD patients

0-normal balance

1-mild loss of balance on arising or with movement, holds onto cage for support

2-major lapse in balance

6. Gross motor skills (upper limb, L/R): partly like the postural instability of PD patients

0-normal, uses limb through a wide range of motion and activities

1-noticeable decrease in capacity to use limb, but used consistently

2-severe decrease in capacity to use limb, rarely used

3-unable or refuses to use limb (including walking)

7. Defense reaction (defensive and/or aggressive response to examiner): not applicable to PD patients

0-normal, reacts appropriately

1-detectable impaired, slowed, abnormal, or shortened response

2-little or no response upon good provocation

Note:

Part A was used to determine the Parkinsonian score [1].

Supplementary Reference

1. Smith RD, Zhang Z, Kurlan R, McDermott M, Gash DM. Developing a stable bilateral model of parkinsonism in rhesus monkeys. Neuroscience 1993, 52: 7–16.
